# Van Wyk–Grumbach Syndrome and Gonadectomy

**DOI:** 10.3390/children11070831

**Published:** 2024-07-08

**Authors:** Abril Adriana Arellano-Llamas, Alvaro Hernandez-Caballero, Efren Delgado-Mendoza, Manuel Alejandro Catalan-Ruiz

**Affiliations:** 1 Instituto Mexicano del Seguro Social, UMAE Hospital de Gineco Obstetricia No. 3 La Raza, Mexico City 02990, Mexico; 2 Instituto Mexicano del Seguro Social, División de Evaluación de Tecnologías en Salud, Mexico City 02990, Mexico; alvheca@yahoo.com; 3 Instituto Mexicano del Seguro Social, UMAE Hospital General La Raza, Mexico City 02990, Mexico; mendoza-efren@hotmail.com (E.D.-M.); catalanruizmanuel@gmail.com (M.A.C.-R.)

**Keywords:** puberty, precocious, ovarian cysts, gonadectomy, Van Wyk–Grumbach syndrome

## Abstract

Van Wyk–Grumbach syndrome (VWGS) refers to the development of peripheral precocious puberty, long-standing hypothyroidism, and gonadal masses; when not diagnosed, an unnecessary gonadectomy may be performed. Herein, we present a case of a 10-year-old girl with Down’s syndrome, short stature, and vitiligo who presented to our hospital with vaginal bleeding and a palpable pelvic mass. Upon ultrasound and topographical examination, bilateral ovarian masses with negative tumor markers were detected. After bilateral gonadectomy, endocrine studies revealed profound hypothyroidism and peripheral puberty that led to the VWGS syndrome diagnosis (TSH 367.3 mUI/mL, isolated menstruation, indetectable LH, and elevated estradiol). Levothyroxine treatment improved obesity and short stature, and sexual hormone replacement began at 13 years of age. The literature on Van Wyk–Grumbach syndrome shows that it presents most often in women, and classic hypothyroidism symptoms always precede the diagnosis. Approximately 11% of patients have Down’s syndrome, sometimes tumor markers are elevated, and some develop severe symptoms (myopathy, short stature, mental delay, ascites, pericardial effusion, Cullen’s sign, pituitary hyperplasia, and severe anemia) that respond to levothyroxine treatment. Conclusions: Children with peripheral precocious puberty and gonadal masses must be studied for hypothyroidism before any radical decision is made.

## 1. Introduction

Unnecessary gonadectomies are frequently performed in girls with benign ovarian masses (21 to 77% of cases), resulting in infertility, osteopenia, and increased cardiovascular risk. Preoperative risk stratification algorithms can reduce the incidence of unnecessary oophorectomies and prevent negative lifelong consequences [1,2].

Hypothyroidism has a significant burden of disease [3]; congenital hypothyroidism affects 1:2000 to 1/4000 newborns, but screening only covers 30% of them worldwide [4,5]. Hypothyroidism symptoms are subtle and insidious; therefore, populations at high risk, such as people with Down’s syndrome, must be screened annually to prevent severe symptoms (short stature, pericardial effusion, muscle injury, dyslipidemia, and mental retardation) [6]. Primary hypothyroidism is caused by the thyroid’s inability to secrete thyroid hormones. It leads through negative feedback to a rise in TSH hormone levels induced by the hypophysis. 

Down’s syndrome affects about 12.59/10,000 to 23.71/10,000 live births [7,8]. Thyroid dysfunction in these patients is common and autoimmune mediated in the majority of cases; the optimal timing to screen for hypothyroidism is controversial, but when symptoms are evident (lethargy, anemia, short stature, and obesity), thyroid function tests are mandatory [9].

In normal circumstances, the gonads activate the follicle maturation process, including the rise in estrogen or testosterone levels and its effects (menstruation or virilization) when follicle-stimulating hormone activates its receptor. Thyroid-stimulating hormone (TSH) can activate the human FSH receptor because glycoproteins in both hormones share a common α-subunit. As a result, hypothyroidism patients may present symptoms related to FSH stimulation, including precocious puberty and ovarian cysts [10].

Van Wyk–Grumbach syndrome is a rare form of hypothyroidism. There is no information about its prevalence, but after an exhaustive search, 54 cases were found in the literature. It is the expression of gonad activation via the elevation of TSH levels. It can include peripheral precocious puberty, gonad enlargement, menstruation, virilization, and, in some cases, pituitary enlargement.

The aim of this paper is to present the case of a girl with Down’s syndrome who underwent bilateral gonadectomy due to benign cysts resulting from untreated chronic primary hypothyroidism. Additionally, we analyzed the literature related to Van Wyk–Grumbach syndrome in other patients.

## 2. Materials and Methods

This study was developed in two stages. The first stage is the presentation of a clinical case; the second stage consists of a review and analysis of the literature. In brief, the terms van Wyk–Grumbach syndrome, precocious puberty, ovarian cyst, children, and hypothyroidism were searched in PubMed, Imbiomed, Google Scholar, and Scopus. We included all articles that describe a Van Wyk syndrome-compatible phenotype. For each clinical case, we describe the sex, age at presentation in the hospital, age at first hypothyroidism sign, the continent of origin, comorbidities, typical hypothyroidism signs, bone age, height in cm, height in standard deviation by CDC charts, TSH concentration at diagnosis, pubertal signs, severe hypothyroidism signs, ultrasound findings, surgical treatment. SPSS V25 was used to analyze the data.

## 3. Results

### 3.1. Case Presentation

A 10-year-old female with Down’s syndrome (karyotype 47XX(20)+21), vitiligo, and a two-month history of vaginal bleeding presented to pediatric consultation with abdominal pain. An abdominopelvic ultrasound revealed an ovarian tumor of 21 cm × 8 cm.

**Figure 1:**
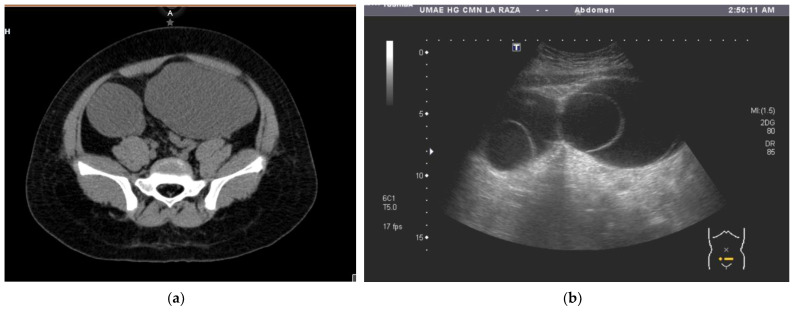
(**a**) Tomography image of the two pelvic tumors, A: Anterior; (**b**) ultrasound image of a pelvic tumor, T: Transductor.

The patient was referred to our hospital, where a second ultrasound found anechoic abdominal lesions with round, dense material inside. A subsequent CT showed two pelvic lesions of 20 HU, one of 174 cc (right ovary) and the other of 333 cc (left ovary). The left one had a round interior image of 250 HU inside. The radiological diagnosis was adnexal vs. mesenteric cystic tumors (Figure 1a,b). Blood tumor markers were negative (Table 1).

A bilateral gonadectomy and salpingectomy were performed on the patient, and the histopathological study found normal salpinges, mucinous cystadenoma, and follicular cysts of the ovaries.

Biochemical tests performed after surgery because of the patient’s Down’s syndrome condition showed severe primary hypothyroidism, LH suppression, elevated estrogen, and hyperlipidemia (Table 1). An endocrine-targeted approach to the patient revealed a history of asthenia, bone pain, progressive weight gain, dry skin one year before surgery, obesity (BMI 59.7 kg/m^2^), short stature (120 cm), and signs of peripheral puberty (Tanner stage 1 in breast and pubic hair and vaginal bleeding).

Finally, Van Wyk–Grumbach syndrome (severe primary hypothyroidism and isosexual peripheral precocious puberty) was diagnosed; she was treated with levothyroxine, and obesity, dry skin, asthenia, and short stature were resolved. Sexual hormone replacement started at the age of 13 years.

### 3.2. Literature Review

We found 54 patients reported to have Van Wyk–Grumbach syndrome [11,12,13,14,15,16,17,18,19,20,21,22,23,24,25,26,27,28,29,30,31,32,33,34,35,36,37,38,39,40,41,42,43,44,45,46,47,48,49,50,51,52,53,54,55,56], with most patients from Asia. Their main characteristics are in Table 2, Table 3, Table 4, Table 5, Table 6, Table 7 and Table 8. The age at diagnosis was from 1.5 to 24 years. All but one case [15] had typical hypothyroidism findings, and short stature was present in more than 60% of the patients. The most common symptoms that led to diagnosis were abdominal pain and vaginal bleeding. Other manifestations were severe anemia [30,31,33,36], ascites [15,31,38], ovarian torsion [16,55], intracranial mass effect [24], and Cullen’s sign [48]. Ultrasound findings included cysts in 80% of cases and complex images in 6.7%. In some cases, tumor markers were measured (31.5%), resulting in positive results in less than one-fifth of the cases (18.5%). Although thyroid substitution can reverse ovarian cysts, seven patients underwent surgery (13%) (11,24,44,51), and in one case, an oophorectomy was avoided just in time [13].

## 4. Discussion

Van Wyk–Grumbach syndrome is caused by peripheral gonadal stimulation mediated by elevated TSH, which results in peripheral precocious puberty. Absent negative feedback by thyroid hormones causes pituitary hyperplasia. Gonadal enlargement and vaginal bleeding in girls can lead to gonadectomy and subsequent lifetime hormonal function loss [22].

The pathophysiology of Van Wyk–Grumbach syndrome remains unclear. It is associated with variable FSH receptor sensitivity and ovarian overstimulation by elevated TSH levels. This phenomenon is seen not only in children but also in adults with recombinant TSH treatment [57,58,59]. In some cases, there are typical FSH receptors. Almost all cases had very high levels of TSH, like in this case.

In almost every case of Grumbach syndrome, classic hypothyroidism symptoms precede precocious puberty, and some symptoms may be severe or life-threatening, such as severe hypercholesterolemia, anemia, intracranial mass effect, myopathy, galactorrhea, or mental retardation. In some reports, ovarian cysts due to hypothyroidism may elevate tumor markers. In this case, short stature and progressive obesity were classical hypothyroidism symptoms. The delayed bone age found in the literature led us to think that a minimal time of TSH exposure is needed to trigger pubertal symptoms.

In all cases, treatment with levothyroxine reversed precocious puberty, ovarian cysts, tumor marker elevation, and dyslipidemia. Typical symptoms of hypothyroidism were also improved, like in this patient, with improvement in obesity and short stature.

As in this case, delayed diagnosis of hypothyroidism results in unnecessary medical visits, tests, and treatments (gonadotrophin tests for precocious puberty, invasive tests for short stature, and surgery). We believe that many cases like ours have not been published [60,61].

Hypothyroidism diagnosis can be a challenge; non-diagnosed congenital hypothyroidism has a 4-year delay in diagnosis even with typical symptoms [62]. It is not unusual to find patients with hypothyroidism with atypical manifestations that have emerged because of a long-standing disease [63]. In particular, growth restriction is found in hypothyroidism, as in our case in 63% of VWG syndrome cases reported in the literature. This is accompanied by a severely delayed bone age, which we believe is directly related to hypothyroidism evolution time [64]. In this case, the evaluation of an endocrinologist after surgery was needed to diagnose the condition, evading the suspicion of the pediatric emergency team and the surgical service.

About 11% of patients with Van Wyk–Grumbach syndrome have Down’s syndrome. Persons living with Down’s syndrome must be screened for thyroid disorders annually, before any surgery or when another autoimmune disease appears. Approximately 50% will develop some form of thyroid dysfunction in their lives (hypothyroidism 39%, congenital hypothyroidism 7%, and hyperthyroidism 3%) [65,66]. The patient presented in this case did not have this annual evaluation, nor was it performed before surgery. This group of patients needs a very diligent pediatric team to prevent this outcome.

Irreversible treatment decisions in children, such as gonadectomy, must be based on reliable tools to avoid overtreatment and its long-term consequences [2].

Children with peripheral precocious puberty and gonadal masses must be studied for hypothyroidism before any radical decision is made, and complete clinical and metabolic evaluation is warranted in the search for other consequences of long-standing hypothyroidism.

## Figures and Tables

**Table 1:** Initial laboratory findings in this case.

Laboratory	Result	Normal Range
Alpha-fetoprotein	4.86 ng/mL	0 to 7 ng/mL
Carcinoembryonic antigen	3.84 ng/mL	0 to 3.8 ng/mL
Chorionic gonadotropin	<0.10	non detectable
CA-125	25.39 UI/mL	0 to 35 UI/mL
TSH	367.3 μUI/mL	0.27 to 4.2 μUI/mL
Total T4	0.49 μg/dL	5.1 a 14.1 μg/dL
Free T4	0.06 ng/dL	0.93 to 1.7 ng/dL
Total T3	<0.20 ng/mL	0.8 to 2 ng/mL
Free T3	1.59 pmol/L	1.73 to 6.3 pmol/L
Anti-thyroglobulin	150.5 ng/dL	5 to 100
LH	<0.10 mUI/mL	<0.10 mUI/mL
Estradiol	63.53 pg/mL	Non detectable
FSH	5.19 mUI/mL	0 to 5 mUI/mL
Total cholesterol	305 mg/dL	120 to 200 mg/dL
Triglycerides	332 mg/dL	35 to 135 mg/dL

**Table 2:** Main characteristics of reports of Van Wyk–Grumbach syndrome.

Variable	Result *n* (%) or * Median (Interquartile Range)
Place of report	
Asia	29 (53.7)
America	18 (33.3)
Europe	7 (13)
Female sex	51(94)
Age at diagnosis, years	9.95 (7.37 to 14) *
Comorbidities	9 (16.6)
Down’s syndrome	6 (11.1)
Alport syndrome	1(1.9)
Hemangioma	1 (1.9)

**Table 3:** Typical hypothyroidism findings at presentation.

Variable	*n* (%) from All Studies or * Median (Interquartile Range)	Percentage of Studies That Report the Symptom
Mental retardation	12 (22.2)	13 (24.1)
Edema/myxedema	18 (33.3)	19 (35.2)
Dry skin	22 (37)	22 (37)
Puffy face	19 (33.3)	19 (33.3)
Hypoactivity or asthenia	23 (38.9)	23 (38.9)
Constipation	13 (24.1)	14 (24.1)
Bradycardia	8 (14.8)	8 (14.8)
Anemia	27 (46.3)	25 (48.1)
Hb g/dL	9 (7.9 to 10) *	
Alopecia	1 (1.9)	1 (1.9)
Irregular menstrual rhythm	6 (11.1)	6 (11.1)
Myopathy or muscular weakness	6 (11.1)	6 (11.1)
Short stature	34 (63)	39 (72)
Height (CDC sz)	−3.47 (−2.45 to −4.76) *	
Obesity	15 (27.8)	17 (31.5)
Hypercholesterolemia	7 (13)	7 (13)
Total cholesterol mg/dL	345 (299 to 444) *	
Delayed bone age	31 (57.4)	36 (64.7)
Bone age delay, years	−3 (−4 to −1.5) *	
Number of findings		
0	1 (1.9)	
1 to 5	35 (64.6)	
6 to 10	18 (33.5)	
Time from typical hypothyroidism findings to diagnosis, years	2 (1 to 5)	
TSH mUI/mL	490 (100 to 939) *	35 (63.3)
TSH > 100	42 (77.8)	
TSH < 100	10 (18.5)	

CDC: Central Disease Center.

**Table 4:** Symptoms that led to medical consultation.

Symptom	*n* (%)
Vaginal bleeding	23 (42.3)
Abdominal pain	15 (27.8)
Abdominal bloating	3 (5.6)
Abnormal menstruation (irregularity or metrorrhagia)	4 (7.4)
Other (ascites, headache, clitoromegaly, Cullen’s sign, abdominal mass, precocious puberty, short stature, and muscular weakness)	9 (16.6)

**Table 5:** Puberal or gonadal-related findings of hypothyroidism at diagnosis.

Symptom	*n* (%)
Puberal or sexual symptom	
Vaginal bleeding	33 (61.1)
Macroorchidism	3 (5.6)
Clitoromegaly	2 (3.7)
Puberal delay	1 (1.9)

**Table 6:** Severe findings of hypothyroidism at diagnosis.

Symptom	*n* (%)
Anemia requiring transfusion	2 (3.8)
Ascites	4 (7.4)
Pericardial effusion	5 (9.2)
Intracranial mass effect	1 (1.9)
Cullen’s sign	1 (1.9)
Ovarian torsion	3 (5.5)
Pituitary hyperplasia	18 (33.3)
Hyperprolactinemia	29 (53.7)
Elevated AST	1 (1.9)

**Table 7:** Gonadal ultrasound findings at diagnosis.

Findings	*n* (%)
Multiple cysts	23 (51.1)
Cysts	13 (28.9)
Ovarian enlargement	5 (11.1)
Ovarian tumor or complex images	3 (6.7)
Other	1 (2.2)

**Table 8:** Surgery indications.

Type of Surgery	*n* (%)
Cystectomy	2 (28.5)
Ovary detorsion	2 (28.5)
Ovary biopsy	1 (14.2)
Bilateral oophorectomy	1 (14.2)
Aborted oophorectomy	1 (14.2)

## Data Availability

Data are contained within the article.
